# Metabolomic analyses of COVID-19 patients unravel stage-dependent and prognostic biomarkers

**DOI:** 10.1038/s41419-021-03540-y

**Published:** 2021-03-11

**Authors:** François-Xavier Danlos, Claudia Grajeda-Iglesias, Sylvère Durand, Allan Sauvat, Mathilde Roumier, Delphine Cantin, Emeline Colomba, Julien Rohmer, Fanny Pommeret, Giulia Baciarello, Christophe Willekens, Marc Vasse, Frank Griscelli, Jean-Eudes Fahrner, Anne-Gaëlle Goubet, Agathe Dubuisson, Lisa Derosa, Nitharsshini Nirmalathasan, Delphine Bredel, Séverine Mouraud, Caroline Pradon, Annabelle Stoclin, Flore Rozenberg, Jérôme Duchemin, Georges Jourdi, Syrine Ellouze, Françoise Levavasseur, Laurence Albigès, Jean-Charles Soria, Fabrice Barlesi, Eric Solary, Fabrice André, Frédéric Pène, Félix Ackerman, Luc Mouthon, Laurence Zitvogel, Aurélien Marabelle, Jean-Marie Michot, Michaela Fontenay, Guido Kroemer

**Affiliations:** 1 INSERM U1015, Gustave Roussy Cancer Campus, 94800 Villejuif, France; 2 Université Paris Saclay, Faculté de Médecine, 94270 Le Kremlin-Bicêtre, France; 3 Metabolomics and Cell Biology Platforms, Gustave Roussy Cancer Campus, 94800 Villejuif, France; 4 INSERM U1138, Gustave Roussy Cancer Campus, 94800 Villejuif, France; 5 Service de Médecine Interne, Hôpital Foch, 92150 Suresnes, France; 6 Service d’Accueil des Urgences, AP-HP, Hôpital Hôtel-Dieu, 75004 Paris, France; 7 Département d’Oncologie Médicale, Gustave Roussy Cancer Campus, 94800 Villejuif, France; 8 Département d’Hématologie, Gustave Roussy Cancer Campus, 94800 Villejuif, France; 9 Service de biologie clinique, Hôpital Foch, 92150 Suresnes, France; 10 Service de virologie, Gustave Roussy Cancer Campus, 94800 Villejuif, France; 11 Centre d’Investigation Clinique – Biothérapie, INSERM CICBT1428, 94800 Villejuif, France; 12 Département de Biologie Médicale, Gustave Roussy Cancer Campus, 94800 Villejuif, France; 13 Département de Réanimation, Gustave Roussy Cancer Campus, 94800 Villejuif, France; 14 Service de Virologie, AP-HP. Centre-Université de Paris, Hôpital Cochin, Paris, France; 15 Service d’Hématologie Biologique, AP-HP, Centre-Université de Paris, Hôpital Cochin, 75014 Paris, France; 16 Université de Paris, Innovative Therapies in Haemostasis, INSERM 1140, F-75006 Paris, France; 17 Université de Paris, Institut Cochin, CNRS UMR8104, INSERM U1016, 75006 Paris, France; 18 Gustave Roussy, Paris-Saclay University, Paris, France; 19 Aix Marseille University, CNRS, INSERM, CRCM, Marseille, France; 20 INSERM U1287, Gustave Roussy Cancer Campus, 94800 Villejuif, France; 21 INSERM U981, Gustave Roussy Cancer Campus, 94800 Villejuif, France; 22 Service de Médecine Intensive et Réanimation, AP-HP, Hôpital Cochin, 75014 Paris, France; 23 Service de Médecine Interne, AP-HP, Hôpital Cochin, 75014 Paris, France; 24 Département d’Innovation Thérapeutique et des Essais Précoces, Gustave Roussy Cancer Campus, 94800 Villejuif, France; 25 Centre de Recherche des Cordeliers, Equipe labellisée par la Ligue contre le cancer, Université de Paris, Sorbonne Université, INSERM U1138, Institut Universitaire de France, Paris, France; 26 Pôle de Biologie, Hôpital Européen Georges Pompidou, AP-HP, 75015 Paris, France; 27 Suzhou Institute for Systems Biology, Chinese Academy of Sciences, Suzhou, China; 28 Department of Women’s and Children’s Health, Karolinska University Hospital, 17176 Stockholm, Sweden

**Keywords:** Metabolomics, Viral infection

## Abstract

The circulating metabolome provides a snapshot of the physiological state of the organism responding to pathogenic challenges. Here we report alterations in the plasma metabolome reflecting the clinical presentation of COVID-19 patients with mild (ambulatory) diseases, moderate disease (radiologically confirmed pneumonitis, hospitalization and oxygen therapy), and critical disease (in intensive care). This analysis revealed major disease- and stage-associated shifts in the metabolome, meaning that at least 77 metabolites including amino acids, lipids, polyamines and sugars, as well as their derivatives, were altered in critical COVID-19 patient’s plasma as compared to mild COVID-19 patients. Among a uniformly moderate cohort of patients who received tocilizumab, only 10 metabolites were different among individuals with a favorable evolution as compared to those who required transfer into the intensive care unit. The elevation of one single metabolite, anthranilic acid, had a poor prognostic value, correlating with the maintenance of high interleukin-10 and -18 levels. Given that products of the kynurenine pathway including anthranilic acid have immunosuppressive properties, we speculate on the therapeutic utility to inhibit the rate-limiting enzymes of this pathway including indoleamine 2,3-dioxygenase and tryptophan 2,3-dioxygenase.

## Introduction

The year 2020 has been overshadowed by coronavirus disease-19 (COVID-19) caused by severe acute respiratory syndrome (SARS) coronavirus-2 (SARS-CoV-2), challenging the resilience of public and private health systems^1^. As a result, COVID-19 is mobilizing an unprecedented technological and scientific effort to diagnose, comprehend, and adequately treat the disease. Indeed, contagion by SARS-CoV-2 provokes a silent or pauci-symptomatic infection in at least 80% of patients, not requiring any treatment^2,3^. However, a substantial fraction of patients with pre-existing and often age-associated medical conditions (obesity, diabetes, hypertension, cardiomyopathy, hematological cancers, and general frailty) develop SARS, requiring hospitalization, oxygen supply, and for the most severe cases mechanical ventilation in the intensive care unit^1,4,5^. Nonetheless, there is a substantial ‘gray zone’, meaning that physically fit and relatively young patients without known pre-existing pathologies may succumb to SARS-CoV-2, calling for the identification of biomarkers that predict COVID-19 severity and help management of patients^1,4,5^.

Beyond genomic studies (to find COVID-19 susceptibility genes)^6^, single-cell transcriptomics performed on circulating leukocytes (to identify inflammatory/ immune cell subsets involved in, and predictive of, COVID-19 pathogenesis)^7,8^ and plasma proteomics (to pinpoint relevant cytokines)^9,10^, metabolomics offers a functional, ‘post-genomic’ characterization of biochemical circuitries influenced by COVID-19 and its treatment. Indeed, a few studies have used mass spectrometric metabolomics to identify COVID-19-induced alterations in circulating metabolites, focusing on the correlation of such parameters with clinical presentation^10^, circulating interleukin (IL)-6 concentrations^11^ or male sex^12^. Additional studies have revealed a metabolomic signature of COVID-19 infection in circulating exosomes^13^ and in the saliva^14^.

Here, we report the results of two metabolomic studies, a first one, non-interventional, in which we correlate shifts in circulating metabolites with the severity stage of COVID-19 patients and a second study, interventional, in which we focus on patients with a uniformly moderate clinical presentation to identify metabolites whose alteration predicts clinical evolution. We identified anthranilic acid, a product of the kynurenine pathway, as a potentially prognostic biomarker of the evolution of COVID-19.

## Results

### COVID-19 stage-dependents shifts in the plasma metabolome

**Fig. 1:**
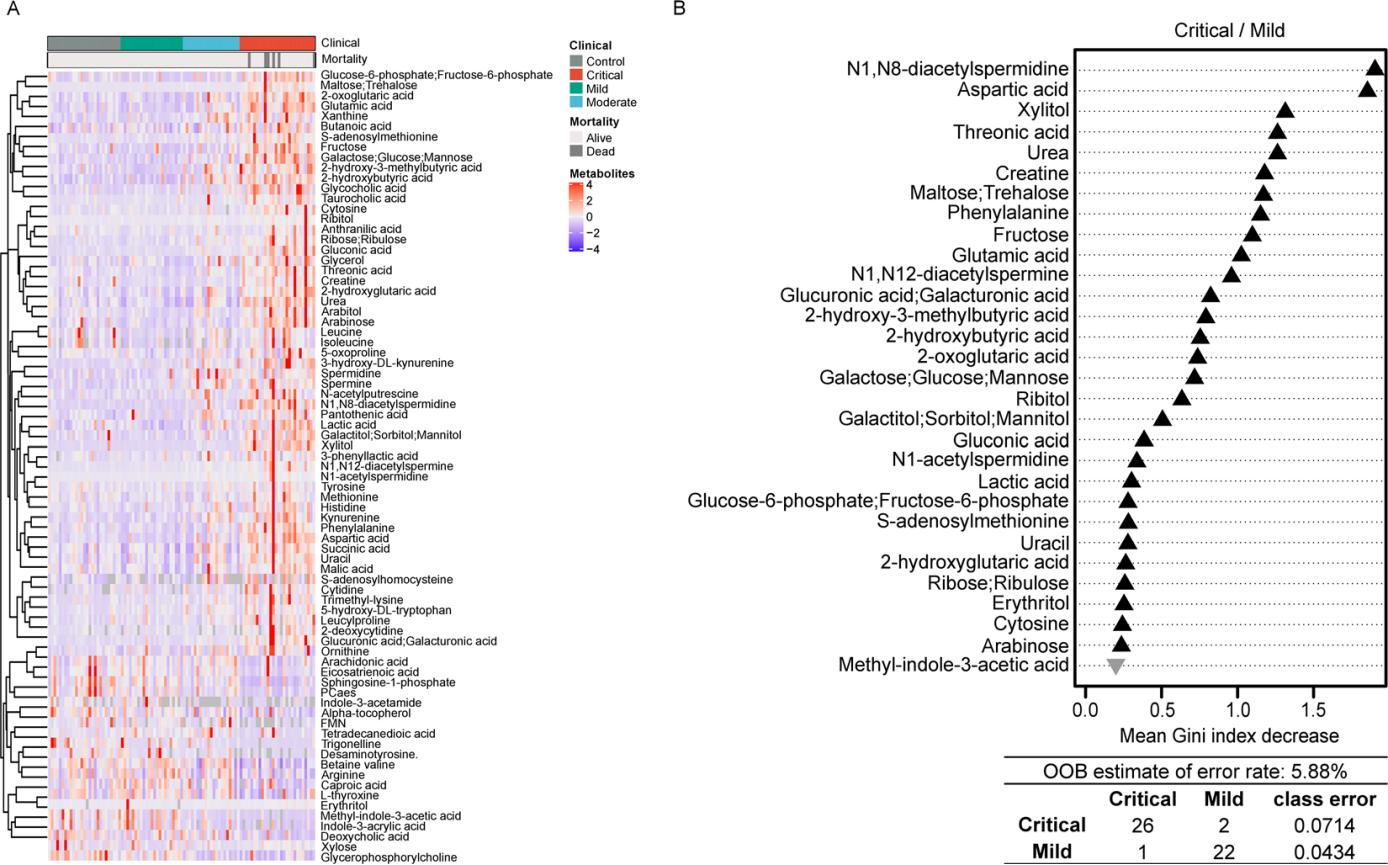
Profound metabolomics alterations associated with COVID-19 clinical severity. **A** Heatmap illustrating the changes in metabolite abundance in the plasma from control (*n* = 27), mild (*n* = 23), moderate (*n* = 21), and critical (*n* = 28) COVID-19 patients. Significant metabolites were identified by Wilcoxon rank-sum test and the false discovery rate (FDR) controlled with Benjamini–Hochberg procedure between patients with critical and mild COVID-19. Hierarchical clustering (Euclidean distance, ward linkage method) of the metabolite abundance is shown. PCaes, total abundance of the different phosphatidylcholines identified in the cohort plasma samples. **B**, Random forest classification model was built using main metabolites altered (*p* < 0.05) between critical and mild COVID-19 patients as a predicting tool. The variables importance (as the mean decrease of the Gini index) for building the model is reported in a dot plot, with dots substituted by an up-pointing triangle to indicate metabolites increased in critical vs mild COVID-19 patients, and by a down-pointing triangle in the opposite case (**B**), the confusion matrix (indicating model accuracy) is depicted below. OOB out-of-bag error.

Targeted and untargeted metabolomics were performed using gas chromatography-mass spectrometry (GC-MS) and ultra-high-pressure liquid chromatography-mass spectrometry (UHPLC-MS) on plasma samples retrieved from a total of 72 patients with PCR-verified diagnosis of SARS-CoV-2 infection and compared to 27 ambulatory patients with flu-like symptoms, negative for SARS-CoV-2. Patients with COVID-19 were staged according to their clinical characteristics into mild (confinement at home, no complementary exams), moderate (standard hospitalization with a radiological diagnosis of pneumonitis, oxygen therapy <9 L/min), and critical (intensive care unit, oxygen therapy >9 L/min) cases. Clustering of mass spectrometry-detectable peaks revealed stage-associated shifts in the metabolome (Supplemental Fig. 1) that become clearly visible upon statistical filtering at *p* < 0.05 (Wilcoxon rank-sum test) and application of a false discovery rate of 0.05 following the Benjamini–Hochberg procedure to identify metabolites which were stringently different between critical and mild COVID-19 patients. Thus, 77 metabolites exhibited stage-dependent alterations in their plasma concentration (Fig. 1A). Among these metabolites, 57 were increased in critical care patients. Random forest classification model was built to rank the metabolites the upregulation or downregulation of which distinguished critical from mild patients (Fig. 1B). Among 30 metabolites, 29 were higher (and 1 lower) in critical than in mild cases, indicating a preponderance of upregulation (*p* < 0.000044, *χ*^2^ analysis).

As described in the literature^3,4^, critical COVID-19 patients were more overweighted, obese, diabetic, and hypertensive than mild COVID-19 and controls patients (Supplemental Table 1). Linear regression was used to control the differences in mean metabolites concentrations between critical and mild COVID-19 patients after adjustment for such comorbidities (Supplemental Table 2).

### Specific changes associated with COVID-19 severity stages

**Fig. 2:**
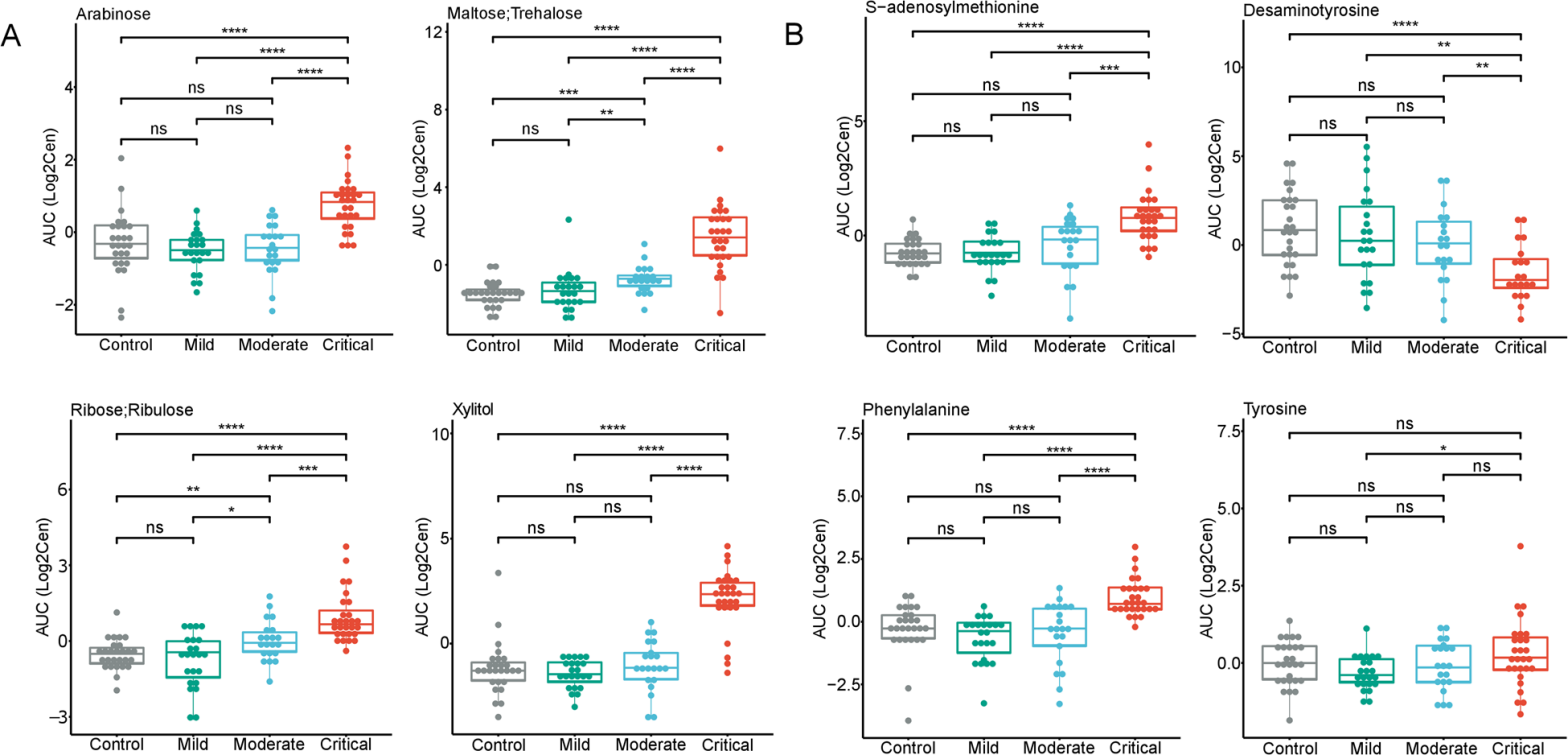
Effects of COVID-19 on circulating sugars and amino acids. Modified carbohydrates (**A**) and amino acids (**B**) were profoundly altered in patients with the most severe COVID-19. Data in **A** and **B** were analyzed by non-parametric unpaired Wilcoxon test (Mann–Whitney) for each two-group comparison. *p < 0.05, **p < 0.01, ***p < 0.001, ****p < 0.0001.

**Fig. 3:**
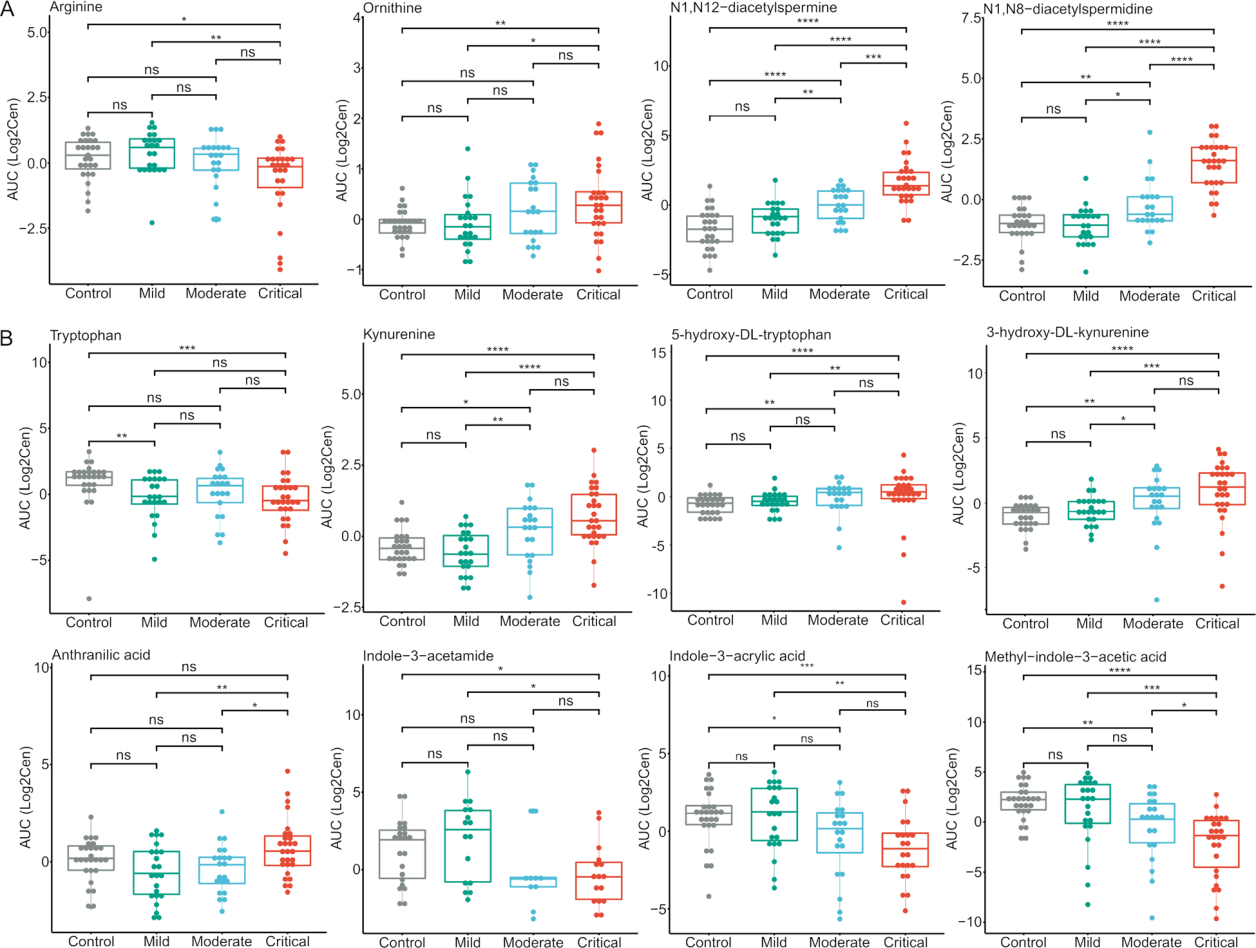
Effecs of COVID-19 on polyamines, tryptophan derivatives and selected amino acids. Polyamines, arginine (**A**) and tryptophane (**B**) pathways alterations in critically ill COVID-19 patients were representative of an immunosuppressive metabolomic state. Data in **A** and **B** were analyzed by non-parametric unpaired Wilcoxon test (Mann–Whitney) for each two-group comparison. *p < 0.05, **p < 0.01, ***p < 0.001, ****p < 0.0001.

A number of simple sugars including arabinose and ribose (and its reduction product ribitol), sugar alcohols (arabitol, erythritol and xylitol), the disaccharide maltose (which is undistinguishable from trehalose), and the trisaccharide raffinose were increased in critical cases (Figs. 1A and 2A and Supplemental Fig. 1A). Moreover, a series of amino acids were elevated in critical care patients: arginine, aspartic acid, glutamic acid, phenylalanine, and tyrosine. In addition, the methylated derivative of lysine, trimethyl-lysine, the methionine derivative (and methyl group donor) S-adenosylmethionine, and the dipeptide leucylproline were elevated (Figs. 1A and 2B), perhaps resulting from increased proteolysis. In contrast, desaminotyrosine was reduced in critical care patients (Figs. 1A and 2B), likely reflecting the use of antibiotics that inhibit the generation of this bacterial metabolite in the gut^15^. One of the few amino acids that decreased with disease severity is arginine, contrasting with an increase in ornithine, spermine, spermidine, and their mono- or diacetylated derivatives (Figs. 1A and 3A), suggesting enhanced polyamine synthesis from arginine. Moreover, tryptophan tended to diminish, while its immunosuppressive metabolite kynurenine increased in critical care patients as compared to mild cases. The kynurenine metabolite anthranilic acid was higher in critical as compared to moderate and mild COVID-19 patients (Figs. 1A and 3B). Of note, the elevation of anthranilic acid has not been found in another study that actually claimed that anthranilic acid decreased in COVID-19 patients as compared to controls^11^. Indeed, we found that another molecule that shared the same neutral monoisotopic mass (137.04768 Da) as anthranilic acid and that decreased in COVID-19 patients (annotated and validated as trigonelline, Supplemental Table 3), perhaps explaining the difference in the results. Since we compared the gas chromatographic retention time of the derivatized analytes to standards (Supplemental Fig. 2), we conclude that anthranilic acid is indeed increased in severe COVID-19. We also found that 3-hydroxy-dl-kynurenine, which his produced from kynurenine by the enzyme kynurenine 3-hydroxylase, and 5-hydroxy-dl-tryptophan, which is produced from tryptophan by the enzyme tryptophan 5-monooxygenase, were increased, correlating with the severity of COVID-19 (Fig. 3B).

Bacterial breakdown products of tryptophan, such as indole, indole-acetamide, indole-3-acrylic acid, and methyl-3-indole-acetate were significantly reduced in critical care patients (Figs. 1 and 3B). Other important metabolic changes affected free fatty acids (arachidonic acid) or carnitine esters, phospholipids, the immunomodulator spingosine-1-phosphate, the secondary bile acid deoxycholic acid, as well as the niacin metabolite trigonelline, that all diminish with disease severity, contrasting with markers of reduced renal clearance (creatine, urea) that increase (Fig. 1A). Altogether, a specific pattern of stage-dependent alterations in the metabolome emerges.

### Prognostic alterations in the circulating metabolome

**Fig. 4:**
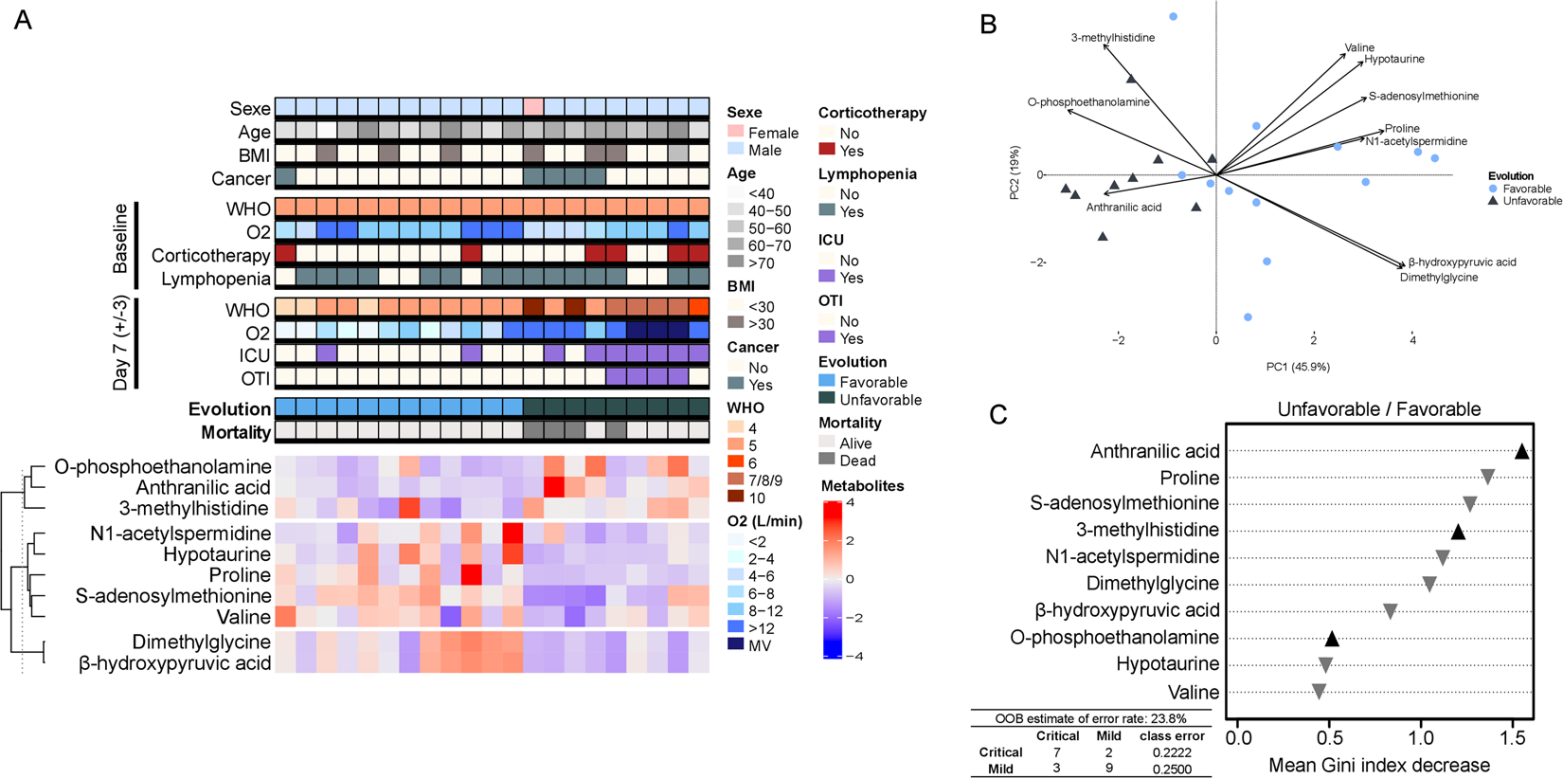
Patients with unfavorable clinical evolution after tocilizumab, infused for worsening pulmonary involvement of COVID-19, had pre-treatment metabolomics differences compared to patients with favorable outcome. **A** Heatmap illustrating pre-tocilizumab metabolite abundance in COVID-19 patients evaluable for clinical evolution after treatment (*n* = 21). Significant metabolites were identified by Wilcoxon rank-sum test between patients with favorable and unfavorable evolution after tocilizumab infusion. BMI Body Mass Index, WHO World Health Organization, O2 oxygenotherapy, ICU intensive care unit, OTI orotracheal intubation. **B** Principal component analysis biplot, showing the contribution of the most significant metabolites (*p* < 0.05) to the discrimination (PC1 45.9%) between patients with favorable and unfavorable evolution after tocilizumab infusion. **C** Random forest classification model was built using main metabolites altered (*p* < 0.05) in baseline samples from COVID-19 patients with favorable and unfavorable evolution after tocilizumab treatment as a predicting tool. The variable importance (as the mean decrease of the Gini index) for building the model is reported in a dot plot, with dots substituted by an up-pointing black triangle to indicate metabolites increased in patients who showed unfavorable vs favorable evolution, and by a gray down-pointing triangle in the opposite case. The confusion matrix (indicating model accuracy) is depicted. OOB out-of-bag error.

The aforementioned results indicate that the progression of COVID-19 is associated with major metabolic shifts, yet do not allow to identify prognostic biomarkers. For this, we recruited a group of 25 patients that were hospitalized in standard conditions (not in the ICU) and were relatively homogeneous in their clinical presentation (Fig. 4A and Supplemental Tables 4 and 5). After the initial determination of their circulating metabolome and the quantitation of serum cytokines (*n* = 21), these patients received standard of care treatments plus tocilizumab. Unfavorable evolution of the COVID-19 (9 out of 21 patients) was defined as a clinical deterioration with WHO progression scale >5, transfer to ICU, mechanical ventilation, or death^16^. Inspection of the global metabolomic profiles did not revealed any major shift that would distinguish the favorable versus unfavorable evolution of COVID-19 neither at baseline nor at day 7 (Supplemental Fig. 3 and Supplemental Tables 6 and 7). Only 10 metabolites were significantly different between patients that demonstrated favorable versus unfavorable evolution (Fig. 4A). However, they allowed a good discrimination between groups (PC1 45.9%) according to their abundance variation (Fig. 4B and Supplemental Fig. 5). To find out the variable importance of these metabolites, we used a random forest classification model. Although the model was limited because of the reduced number of individuals in the study, it showed that, among the most significant metabolites, the upregulation of anthranilic acid coupled to the diminution of S-adenosylmethionine and proline stood out as parameters that allowed to distinguish the unfavorable and favorable evolution of COVID-19 patients respectively (Fig. 4C). Dimethylglycine, ß-hydroxypyruvate, N1-acetylspermidine, hypotaurine, and valine were significantly lower in patients with an unfavorable evolution, while 3-methylhistidine and O-phosphoethanolamine were higher, but these changes had a lower impact according to the random forest classification (Fig. 4B).

### Prognostic immunometabolic correlations

**Fig. 5:**
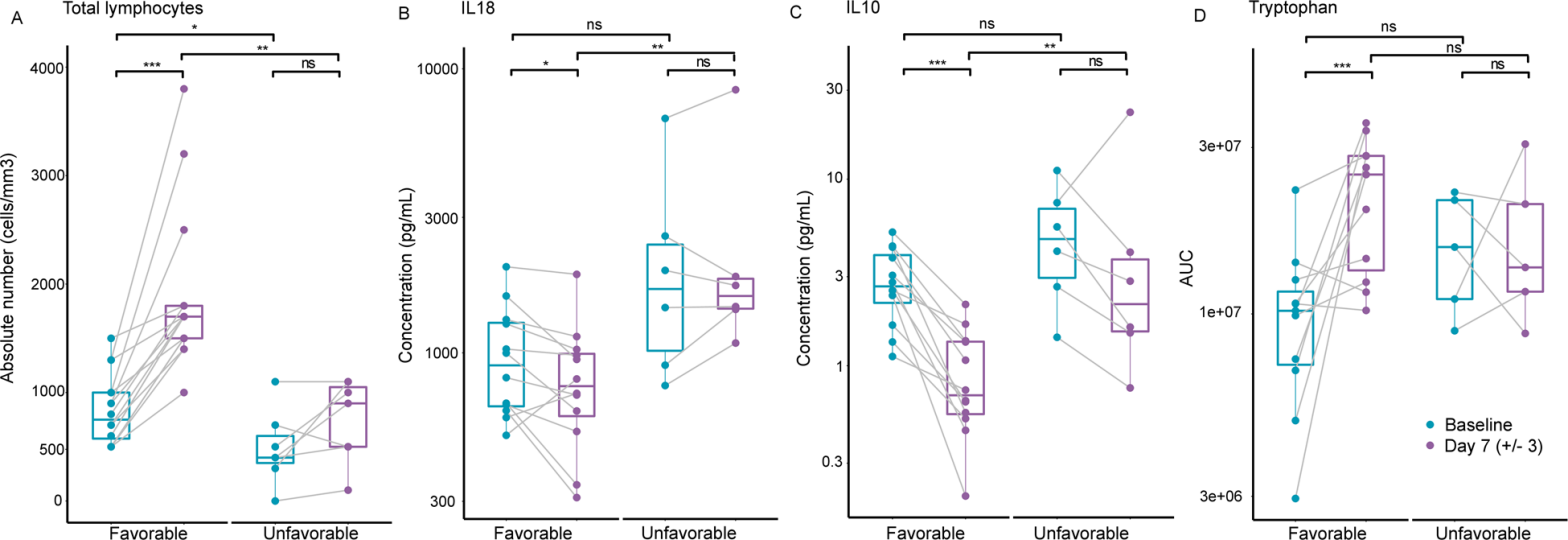
COVID-19 Patients with unfavorable outcome after tocilizumab infusion did not improved lymphopenia, inflammatory, immunosuppressive, and metabolomic abnormalities instead of patients who evolved towards clinical improvement. Patients with paired baseline and post treatment (day 7 ± 3) serum samples are represented (*n* = 18). The measured parameters include total lymphocyte counts (**A**) as well as the concentrations of IL18 (**B**), IL10 (**C**) and tryptophan (**D**). Wilcoxon signed-rank test was used to compare paired baseline and post treatment measures and Wilcoxon rank-sum test to compare baseline or day 7 (±3) measures between patients with response or no response. *p < 0.05, **p < 0.01, ***p < 0.001, ****p < 0.0001.

**Fig. 6:**
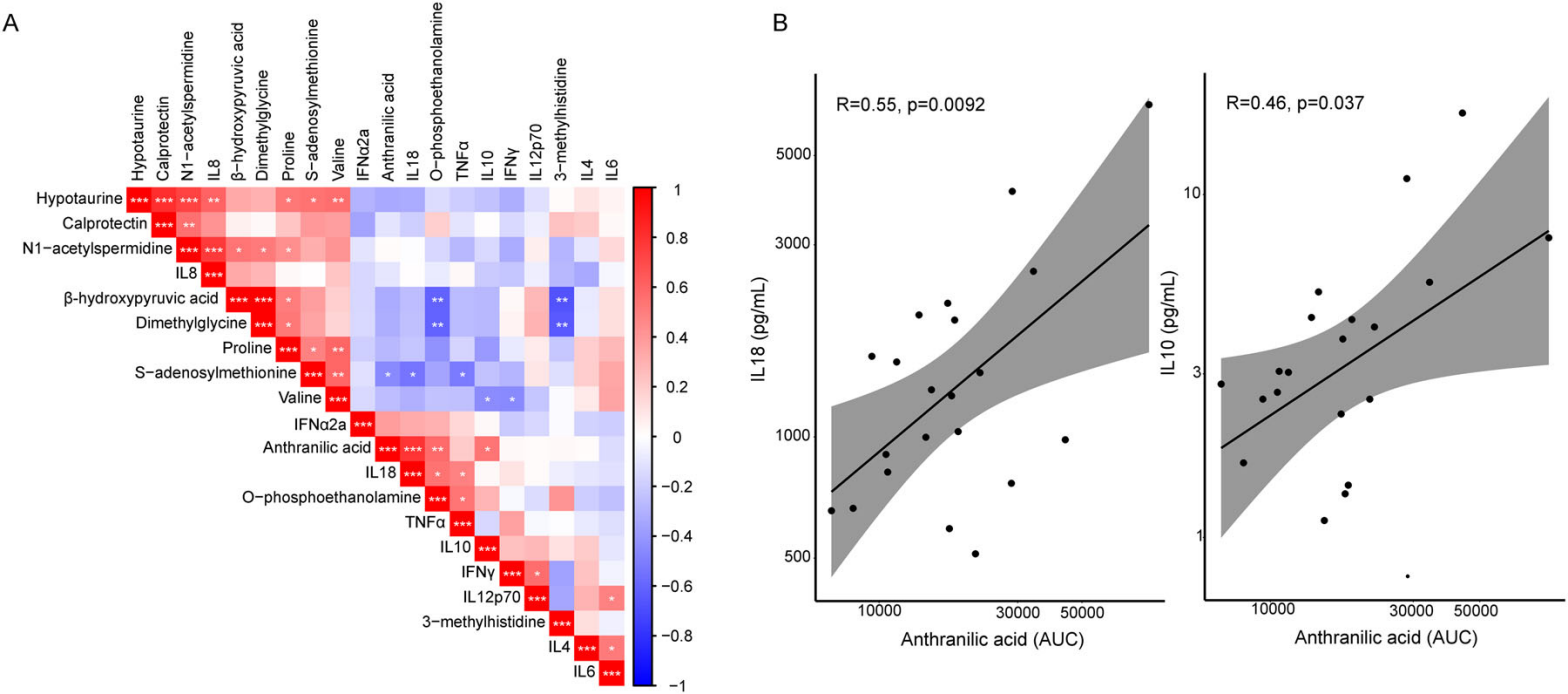
Correlations between cytokines and metabolites before tocilizumab infusion in patients with worsening COVID-19 highlighted that dysregulated metabolomic and immunologic pathways were closely related to clinical worsening of patients developing critical COVID-19. **A** Correlation between cytokines and most significant metabolites at baseline was analyzed by Pearson correlation. *p < 0.05, **p < 0.01, ***p < 0.001. **B** Pearson correlations between IL10 and IL18 with anthranilic acid serum levels.

None of the 10 cytokines measured at baseline did exhibit significant differences between the patients with favorable and unfavorable clinical evolution (Supplemental Fig. 4), in line with the similar clinical presentation of the patients. At difference with patients that exhibited an unfavorable evolution, patients who ameliorated their condition exhibited an increase in total lymphocyte counts (Fig. 5A), a decrease in the inflammatory cytokine interleukin (IL)-18 (IL18) (Fig. 5B), a reduction in the immunosuppressive factor IL10 (Fig. 5C) and an increase in circulating tryptophan levels (Fig. 5D). Correlation plots revealed a median correlation (all values positive) among cytokines of 0.1573, between cytokines and metabolites of 0.2116, and among metabolites of 0.3702 (which was significantly higher than the intragroup correlation and the correlation among cytokines, *p* < 0.0001, Mann–Whitney test), suggesting a more robust coordination of metabolic as compared to inflammatory pathways (Fig. 6A). IL8 correlated with N1-acetylspermidine and hypotaurine, tumor necrosis factor-α (TNFα) with O-phosphoethanolamine (Fig. 6A), and anthranilic acid with both IL10 and IL18 (Fig. 6B) at baseline, before the initiation of the treatment. This latter correlation appears particularly intriguing because anthranilic acid is ranked as the best negative prognostic marker (Fig. 4B) and both IL10 and IL18 remain elevated in the context of an unfavorable evolution (Fig. 5).

## Discussion

The present study has been designed to unravel COVID-19 stage-dependent and prognostic alterations in the circulating metabolome. Strong shifts across multiple classes of metabolites were observed among different stages of COVID-19, from mild through moderate to critical disease. These shifts reflect in part iatrogenic effects such as the apparent improvement of the nutritional state (with higher levels of circulating sugars but lower levels of free fatty acids and ketone bodies, which would be indicative of acute undernutrition) in the critical stage and the reduction of bacterial metabolites (such as the tyrosine metabolite desaminotyrosine and the tryptophane metabolites indole, indole-3-acetamide, indole-3-acrylic acid, and methyl-3-indole-acetate), likely resulting from the administration of broad-spectrum antibiotics. Other metabolomic shifts may reflect proteolysis (with an increase in free amino acids and amino acid derivatives), as well as ongoing organ failure affecting the kidney (enhanced acetyl polyamine, creatine, and urate levels) and the liver (reduced primary bile acid production).

Most intriguingly, however, COVID-19 appears to be associated with metabolic signs of immunosuppression, as indicated by the increase of kynurenic acid and anthranilic acid. Tryptophan was diminished in mild and critical COVID-19 patients compared to uninfected controls, suggesting a disease-associated activation of tryptophan-consuming indoleamine 2,3-dioxygenase (IDO) and tryptophan 2,3-dioxygenase (TDO) that produce the kynurenic acid precursor kynurenine^17^. Anthranilate is a downstream metabolite of kynurenine^18^, with marked immunosuppressive effects^19^. Previous work has identified an activation of the kynurenine pathway (though without an elevation of anthranilate) in COVID-19 patients, correlating with an elevation of IL6 levels^11^, which in turn are associated with poor prognosis^20–23^.

Small, but specific differences were observed in a cohort of patients that demonstrated a similar clinical stage at presentation, but dissimilar evolution during hospitalization. Some metabolites that apparently were not COVID-19 stage-associated were different between patients that demonstrated a favorable or unfavorable evolution. This applies to dimethylglycine, 3-methylhistidine and O-phosphoethanolamine, proline, and valine. Some metabolites exhibited a behavior that can be classified as ‘paradoxical’. Thus, N1-acetylspermidine, S-adenosylmethionine, and hypotaurine that are highest among severe COVID-19 patients are associated with favorable prognosis, perhaps because their production reflects an attempt to attenuate the pathogenesis of COVID-19. Indeed, in preclinical models, the N1-acetylspermidine precursor, spermidine, has marked anti-inflammatory and immunostimulatory effects^24–27^. Administration of S-adenosylmethionine attenuates the cytokine storm induced by bacterial sepsis^28^ and mediates immunostimulatory effects in a cancer model^29^. Clinical trials have demonstrated that taurine, the downstream metabolite of hypotaurine, decreases serum markers of inflammation including C-reactive protein^30^, which is a negative prognostic marker of COVID-19^31^.

In sharp contrast to these ‘paradoxical’ associations, one metabolite exhibited a ‘concordant’ behavior. This applies to anthranilic acid, the concentration of which increases with disease severity and which also predicts unfavorable prognosis. This observation places the kynurenine pathway in the limelight of this study. Larger prospective studies are required to validate the conjecture that metabolomic profiling and specific measurement of selected metabolites including anthranilic acid may predict the fate of COVID-19 patients. Circulating anthranilic acid levels reportedly correlate with hyperleptinemia in schizophrenia^32^ and are increased in the plasma of patients with type-1 (but not type-2) diabetes^33^ and subgroups of patients with chronic liver disease^34^, calling for additional investigations of possible confounding factors. Irrespective of these considerations, it might be worthwhile to explore the experimental treatment of COVID-19 with IDO and TDO inhibitors that are in clinical development^35–38^. Such inhibitors have been generated as immune checkpoint inhibitors for the treatment of cancer, but have not yet received regulatory approval. The fact that high levels of anthranilic acid predict the maintenance of high levels of IL10 and IL18 suggests (but does not prove) that the kynurenine pathway has an immunomodulatory impact on COVID-19 pathogenesis. However, this speculation should be tested by treating anthranilic acid-high COVID-19 patients with IDO/TDO or other kynurenine pathway inhibitors within a dedicated Phase 2 clinical trial.

## Methods

### Patients

All patients were recruited by different hospitals of the Assistance Publique Hôpitaux de Paris (AP-HP) network or at Foch Hospital or Gustave Roussy. The non-interventional study was approved by institutional review boards (IRB) of Cochin-Port Royal (Paris, France) hospital and the ethical committee of Cochin-Port Royal Hospital (CLEP Decision N: AAA-2020-08023), and conformed to the principles outlined in the Declaration of Helsinki. Controls (*n* = 29) were symptomatic patients who were seen at the Hôtel-Dieu screening unit and were negative for SARS-CoV-2 RT-PCR on pharyngeal swab. Mild COVID-19 patients (*n* = 23) were defined by having limited clinical symptoms (fever, cough, diarrhea, myalgia, and anosmia/ageusia) that did not require CT scan or hospitalization. Moderate cases (*n* = 21) were defined as symptomatic patients with dyspnea and radiological findings of pneumonia on thoracic CT scan, requiring hospitalization and a maximum of 9 L/min of oxygen. Critical patients (*n* = 28) were those hospitalized in the ICU with respiratory distress requiring 10 L/min of oxygen or more, without or with endotracheal intubation and mechanical ventilation. “Comorbidities” variable for adjustment was considered for patients with obesity or diabetes or chronic kidney disease and hypertension.

The interventional study was approved by the Foch IRB (approval number IRB00012437) and was registered on the National Institute of Health data platform INDS (no 4710280420). Patients received tocilizumab, in an off-label use setting, to treat severe COVID-19, at Gustave Roussy and Foch Hospital, over the period of March 20 and 5 April 2020. Inclusions criteria were: (i) Patients who received at least one dose of tocilizumab, as treatment of COVID-19. (ii) ≥18 years, informed, and not opposed for retrospective use of their anonymized health care files. (iii) Diagnosis of COVID-19 confirmed by RT-PCR test, with respiratory symptoms, shortness of breath and requirement of oxygen therapy and pulmonary images compatible with COVID-19. (iv) Patients at risk of developing respiratory distress due to COVID-19, with worsening of oxygen therapy supplementation equal or more than 4 L/min and requirement by increase of >50% of the need for supplemental oxygen therapy in the last 24 h before first dose of tocilizumab. Exclusion criteria for evaluable population for the response to tocilizumab were: (i) Patients placed under mechanical ventilation with intubation due to the COVID-19 before the first dose of tocilizumab treatment. (ii) Respiratory failure related to other cause than COVID-19 at tocilizumab initiation. (iii) Patients, who have previously received anti-IL6 receptor therapy in the last 3 weeks before tocilizumab initiation. (iii) Alanine transaminase/aspartate transaminase (ALT/AST) >5 times the upper limit of normal at timing of first dose of tocilizumab. (iv) Absolute neutrophil count <1.0 × 10^9^ or platelets <50 × 10^9^ at timing of first dose of tocilizumab. Tocilizumab was given intravenously at 8 mg/kg and could be repeated once in the following 48 h if necessary. All patients were followed until day 30 after the first dose of tocilizumab. Patients’ sera were collected and stored before and after treatment. Favorable clinical evolution after tocilizumab infusion was retained in patients evaluable for the outcome and fulfilling the following three criteria. Criterion 1: on day 14 post first dose of tocilizumab, the patient has a WHO progression scale ≤ 5^16^. Criterion 2: between days 1 and 14 after the first dose of tocilizumab, the patient is alive and did not need to have at any time recourse to invasive mechanical ventilation (orotracheal intubation) and without any new intention of “non-realization of resuscitation or ventilation”. Criterion 3: the respiratory symptoms related to COVID-19 clinically significantly improved with decrease in oxygen requirements after first dose of tocilizumab and the WHO scale did not deteriorate after the administration of the first dose of tocilizumab.

### Sampling

Human peripheral blood from the first cohort was collected into sterile dry vacutainer tubes with 3.2% buffered sodium citrate solution. Samples were centrifuged twice (2500 × *g*/20 min), and plasma was collected. Regarding the samples from the interventional study, human peripheral blood was collected into sterile dry vacutainer tubes and centrifuged (1500–2000 × *g*/15 min) for serum collection. Fifty microliters of sample were mixed with 500 µL of a cold solvent mixture (meOH/water, 9/1, −20 °C, with a cocktail of internal standards), vortexed and centrifuged (10 min at 1500 × *g*, 4 °C) for metabolite extraction and protein precipitation. The supernatants were collected, split in 4 fractions, and treated according to the protocols described previously^39^. Briefly, 2 fractions of 120 µL each (1st and 2nd fractions, respectively) of sample extract were transferred to an injection amber glass vial (with fused-in insert) and evaporated to dryness (Techne DB3, Staffordshire, UK) at 40 °C. The 1st dried fraction was solubilized in 50 µL of methoxyamine (CAS 593-56-6; 20 mg/mL in pyridine, Sigma-Aldrich), and left to incubate overnight, at room temperature and protected from light. The next day, derivatization was carried out by adding 80 µL of MSTFA (CAS 24589-78-4; Supelco), followed by 30 min-incubation at 40 °C. Derivatized samples were immediately used for GC/MS injection and analysis. The 2nd dried fraction was recovered with 100 µL of ultra-pure water and kept at −80 °C until injection and analysis by UHPLC/MS. The 3rd fraction consisted of 40 µL of sample extract transferred to an injection amber glass vial (with fused-in insert) for derivatization and SCFA analysis. Sample derivatization was carried out by adding 20 µL of 3-NPH (200 mM in meOH; CAS 636-95-3; Sigma-Aldrich) and 20 µL of EDC (150 mM in meOH; CAS 25952-53-8; Sigma-Aldrich) to the sample. Immediately after incubation (1 h/ 40 °C), 80 µL of water were added, and the derivatized samples were used for UHPLC/MS injection and analysis. Finally, the 4th fraction together with the sample pellet were re-extracted with 80 µL of 2% SSA (in meOH), vortexed and centrifuged (10 min at 15,000 × *g*, 4 °C). The supernatant (200 µl) was transferred to an injection polypropylene vial (with fused-in insert) and evaporated to dryness (Techne DB3, Staffordshire, UK) at 40 °C. Dried samples were recovered with 200 µl of ultra-pure water and kept at −80 °C until injection and analysis by UHPLC/MS for polyamines detection.

### Cytokine measurements

Serum samples were monitored using the V-plex Proinflammatory panel 1 Human kit (Meso Scale Discovery, ref: K15049D) according to the manufacturer’s instructions, for the measurement of IFNγ, IL1β, IL2, IL4, IL6, IL8, IL10, IL12p70, IL13, and TNFα. Soluble Calprotectin (diluted 1:100), IFNα2a and IL18 were analyzed using a R-plex Human Calprotectin Antibody Set (Meso Scale Discovery, ref: F21YB), the ultra-sensitive assay S-plex Human IFNa2a kit (Meso Scale Discovery, ref: K151P3S) and the U-plex Human IL18 assay (Meso Scale Discovery, ref: K151VJK), respectively, following manufacturer’s instructions. Acquisitions and analyses of soluble cytokines and calprotectin were performed on a MESO QuickPlex SQ120 reader and the MSD’s Discovery Workbench 4.0. Each serum sample was assayed twice with the average value taken as the result.

### Widely targeted analysis of intracellular metabolites

#### GC/MS

Derivatized samples for GC/MS analysis (1st fraction) were injected (1 µL) into a gas chromatograph (Agilent 7890B; Agilent Technologies, Waldbronn, Germany) coupled to a triple quadrupole mass spectrometer (QQQ/MS; 7000C Agilent Technologies, Waldbronn, Germany), equipped with a high sensitivity electronic impact source (EI) operating in positive mode. Injection was performed in splitless mode. Front inlet temperature was kept at 250 °C, transfer line and ion-source temperature were 250 °C and 230 °C, respectively. Septum purge flow was fixed at 3 mL/min, purge flow to split vent operated at 80 mL/min during 1 min and gas saver mode was set to 15 mL/min after 5 min. Helium gas flowed through column (HP-5MS, 30 m × 0.25 mm, i.d. 0.25 mm, d.f. J&WScientific, Agilent Technologies Inc.) at 1 mL/min. Column temperature was held at 60 °C for 1 min, raised to 210 °C (10 °C/min), then to 230 °C (5 °C/min), to finally reach 325 °C (15 °C/min), and hold at during 5 min. Collision gas was nitrogen.

#### UHPLC/MS

Targeted UHPLC/MS analyses were performed on a RRLC 1260 system (Agilent Technologies, Waldbronn, Germany), with an autosampler kept at 4 °C, and a pelletier oven for rigorous control of the column temperature. The UHPLC was coupled to a QQQ/MS 6410 (Agilent Technologies) equipped with an electrospray source, using nitrogen as collision gas. For bile acids detection, 10 µL from samples recovered in water (2nd fraction) were injected into a Poroshell 120 EC-C8 (100 mm × 2.1 mm particle size 2.7 µm; Agilent technologies) column protected by a guard column (XDB-C18, 5 mm × 2.1 mm particle size 1.8 μm). Mobile phase consisted of 0.2% formic acid (A) and ACN/IPA (1/1; v/v) (B) freshly made. Flow rate was set to 0.3 mL/min, and gradient as follow: 30% B during 1.5 min; increased to 60% B over 9 min; and finally to 98% B for 2 minutes (column washing), followed by 2 min of column equilibration at 30% B (initial conditions). After each injection, needle was washed twice with IPA and thrice with water. The QQQ/MS was operated in negative mode. Gas temperature and flow were set to 325 °C and 12 L/min, respectively. Capillary voltage was set to 4.5 kV.

Derivatized samples for SCFA detection (3rd fraction) were injected (10 μL) into a Zorbax Eclipse XBD C18 (100 mm × 2.1 mm particle size 1.8 µm; Agilent technologies) column, protected by a guard column (XDB-C18, 5 mm × 2.1 mm particle size 1.8 μm). Column oven maintained at 50 °C during analysis. Mobile phase consisted of 0.01% formic acid (A) and ACN (0.01% formic acid) (B). Flow rate was set to 0.4 mL/min, and gradient as follow: 20% B during 6 min; increased to 45% B over 7 min; and finally to 95% B for 5 minutes (column washing), followed by column equilibration phase at 20% B, during 4 min. The QQQ/MS was operated in negative mode. Gas temperature was set to 350 °C with a gas flow of 12 L/min. Capillary voltage was set to 4.0 kV.

Polyamines were detected in the 4th fraction after injection of 10 μL of sample were into a Kinetex C18 (150 mm × 2.1 mm particle size 2.6 µm; Phenomenex) column protected by a guard column C18 (5 mm × 2.1 mm, particle size 1.8 μm). Column oven maintained at 40 °C during analysis. The gradient mobile phase consisted of 0.1% HFBA (Sigma-Aldrich) (A) and ACN (0.1% HFBA) (B) freshly made. The flow rate was set to 0.2 ml/min, and gradient as follow: from 5% (initial conditions) to 40% B in 10 min; then 90% B maintained 2.5 min, and finally equilibration to initial conditions, 5% B, for 4 min. The QQQ/MS was operated in positive mode. The gas temperature was set to 350 °C with a gas flow of 12 l/min. The capillary voltage was set to 3.5 kV. At the end of each UHPLC/MS batch analysis, column was rinsed with 0.3 mL/min of ultra-pure water (A) and ACN (B) as follow: 10% B during 20 min, to 90% B in 20 min, and maintained during 20 min before shutdown. MRM scan mode was used for targeted analysis in both GC and UHPLC/MS. Peak detection and integration were performed using the Agilent Mass Hunter quantitative software (B.07.01).

### Pseudo-targeted analysis of intracellular metabolites

The profiling analysis was performed with a Dionex Ultimate 3000 UHPLC system (Thermo Scientific) coupled to an Orbitrap mass spectrometer (q-Exactive, Thermo Fisher Scientific) equipped with an electrospray source operating in both positive and negative mode, and acquired samples in full scan analysis mode, from 100 to 1200 m/z. LC separation was performed on reversed phase (Zorbax Sb-Aq 100 ×2.1 mm × 1.8 µm particle size), with mobile phases: 0.2% acetic acid (A) and ACN (B). Column oven was kept at 40 °C. Ten microliters of aqueous sample (2nd fraction) were injected for metabolite separation with a gradient starting from 2% B, increased to 95% B in 22 min, and maintained during 2 min for column rinsing, followed by column equilibration at 2% B for 4 min. Flow rate was set to 0.3 mL/min. The q-Exactive parameters were: sheath gas flow rate 55 au, auxiliary gas flow rate 15 au, spray voltage 3.3 kV, capillary temperature 300 °C, and S-Lens RF level 55V. The mass spectrometer was calibrated with sodium acetate solution dedicated to low mass calibration. Data were treated by the quantitative node of Thermo Xcalibur^TM^ (version 2.2) in a pseudo-targeted approach with a home-based metabolites list.

### Untargeted analysis of intracellular metabolites

Raw data files obtained by the previously described pseudo-targeted analysis were also used to perform unbiased profiling analysis, using the Thermo Compound Discoverer (3.1.). After sample injection and data acquisition, raw data files were processed following a customized node-based workflow for identifying unknown compounds in metabolomics. Spectra selection and retention time alignment were performed, followed by removal of background noise and baseline correction. Next, the processing workflow found chromatographic peaks for unknown compounds (molecular weight, MW, x retention time, RT) extracting all relevant spectral and chromatographic information, to predict the elemental composition of the unknowns. All data was exported to R software (version 3.4) for data representation.

### Statistical analysis

All targeted and pseudo-targeted treated data were merged and cleaned with a dedicated R (version 3.4) package (@Github/Kroemerlab/GRMeta). Calculations and statistical tests were performed using R v3.4. Wilcoxon-Mann–Whitney test was used to assess differences in concentration between two different groups. When indicated, the false discovery rate (FDR, *p* > 0.05) was controlled using the Benjamini–Hochberg procedure. Data representation was performed with softwares R v3.6 and Rstudio v1.2.1335 using tidyverse, dplyr, ggplot2, ggpubr, complexheatmap, and corrplot packages. Principal component analysis biplot was built using FactoMineR and factoextra packages, after selection of the metabolites significantly different (*p* < 0.05) between clinical evolution groups (“unfavorable” and “favorable”), at baseline. Data were scaled unit variance before the analysis.

### Determination of most discriminating metabolites with Random Forest Classification Model

Selected metabolites were thereafter used for training a random forest classification model using the R *caret* package. This machine learning tool allowed to classify the relative importance of metabolites for distinguishing COVID-19 stage (here classified in a binary fashion as «critical» and «mild») and clinical evolution (“unfavorable” and “favorable”), by computing the mean decrease of the Gini index (an entropy-like measure of the impurity) over the random forest nodes that were split on them.

### Supplementary information

Supplementary Figure Legends

Supplementary figure 1

Supplementary figure 2

Supplementary figure 3

Supplementary figure 4

Supplementary figure 5

Supplementary Table 1

Supplementary Table 2

Supplementary Table 3

Supplementary Table 4

Supplementary Table 5

Supplementary Table 6

Supplementary Table 7
