# Correction: Metabolomic analyses of COVID-19 patients unravel stage-dependent and prognostic biomarkers

**DOI:** 10.1038/s41419-024-06519-7

**Published:** 2024-02-14

**Authors:** François-Xavier Danlos, Claudia Grajeda-Iglesias, Sylvère Durand, Allan Sauvat, Mathilde Roumier, Delphine Cantin, Emeline Colomba, Julien Rohmer, Fanny Pommeret, Giulia Baciarello, Christophe Willekens, Marc Vasse, Frank Griscelli, Jean-Eudes Fahrner, Anne-Gaëlle Goubet, Agathe Dubuisson, Lisa Derosa, Nitharsshini Nirmalathasan, Delphine Bredel, Séverine Mouraud, Caroline Pradon, Annabelle Stoclin, Flore Rozenberg, Jérôme Duchemin, Georges Jourdi, Syrine Ellouze, Françoise Levavasseur, Laurence Albigès, Jean-Charles Soria, Fabrice Barlesi, Eric Solary, Fabrice André, Frédéric Pène, Félix Ackerman, Luc Mouthon, Laurence Zitvogel, Aurélien Marabelle, Jean-Marie Michot, Michaela Fontenay, Guido Kroemer

**Affiliations:** 1 INSERM U1015, Gustave Roussy Cancer Campus, 94800 Villejuif, France; 2 Université Paris Saclay, Faculté de Médecine, 94270 Le Kremlin-Bicêtre, France; 3 Metabolomics and Cell Biology Platforms, Gustave Roussy Cancer Campus, 94800 Villejuif, France; 4 INSERM U1138, Gustave Roussy Cancer Campus, 94800 Villejuif, France; 5 Service de Médecine Interne, Hôpital Foch, 92150 Suresnes, France; 6 Service d’Accueil des Urgences, AP-HP, Hôpital Hôtel-Dieu, 75004 Paris, France; 7 Département d’Oncologie Médicale, Gustave Roussy Cancer Campus, 94800 Villejuif, France; 8 Département d’Hématologie, Gustave Roussy Cancer Campus, 94800 Villejuif, France; 9 Service de biologie clinique, Hôpital Foch, 92150 Suresnes, France; 10 Service de virologie, Gustave Roussy Cancer Campus, 94800 Villejuif, France; 11 Centre d’Investigation Clinique – Biothérapie, INSERM CICBT1428, 94800 Villejuif, France; 12 Département de Biologie Médicale, Gustave Roussy Cancer Campus, 94800 Villejuif, France; 13 Département de Réanimation, Gustave Roussy Cancer Campus, 94800 Villejuif, France; 14 Service de Virologie, AP-HP. Centre-Université de Paris, Hôpital Cochin, Paris, France; 15 Service d’Hématologie Biologique, AP-HP, Centre-Université de Paris, Hôpital Cochin, 75014 Paris, France; 16 Université de Paris, Innovative Therapies in Haemostasis, INSERM 1140, F-75006 Paris, France; 17 Université de Paris, Institut Cochin, CNRS UMR8104, INSERM U1016, 75006 Paris, France; 18 Gustave Roussy, Paris-Saclay University, Paris, France; 19 Aix Marseille University, CNRS, INSERM, CRCM, Marseille, France; 20 INSERM U1287, Gustave Roussy Cancer Campus, 94800 Villejuif, France; 21 INSERM U981, Gustave Roussy Cancer Campus, 94800 Villejuif, France; 22 Service de Médecine Intensive et Réanimation, AP-HP, Hôpital Cochin, 75014 Paris, France; 23 Service de Médecine Interne, AP-HP, Hôpital Cochin, 75014 Paris, France; 24 Département d’Innovation Thérapeutique et des Essais Précoces, Gustave Roussy Cancer Campus, 94800 Villejuif, France; 25 Centre de Recherche des Cordeliers, Equipe labellisée par la Ligue contre le cancer, Université de Paris, Sorbonne Université, INSERM U1138, Institut Universitaire de France, Paris, France; 26 Pôle de Biologie, Hôpital Européen Georges Pompidou, AP-HP, 75015 Paris, France; 27 Suzhou Institute for Systems Biology, Chinese Academy of Sciences, Suzhou, China; 28 Department of Women’s and Children’s Health, Karolinska University Hospital, 17176 Stockholm, Sweden

**Keywords:** Metabolomics, Viral infection

Correction to: *Cell Death and Disease* (2021) 10.1038/s41419-021-03540-y, published online 11 March 2021

In this article the Funding section has been added:

“European Union Horizon 2020 research project Oncobiome (grant number 825410)”

The original article has been corrected.

