# Supplementary material for: Metabolomic analyses of COVID-19 patients unravel stage-dependent and prognostic biomarkers
Source: Cell Death Dis. 2021 Mar 11;12(3):258. doi: 10.1038/s41419-021-03540-y (PMC7948172; doi:10.1038/s41419-021-03540-y)
Supplement: Supplementary file 1 — Supplementary Figure Legends [file 41419_2021_3540_MOESM1_ESM.docx]

**Legends to supplementary figures**

**Supplemental Figure 1. Heatmap showing the abundance changes of all the identified metabolites in COVID-19 patients plasma samples from the non-interventional study.** Metabolic signature obtained through targeted (A) and untargeted metabolomic analysis (B). Targeted analysis allowed the identification of 223 metabolites in the plasma from control (n=27), mild (n=23), moderate (n=21) and critical (n=28) COVID-19 patients, while over 17000 metabolic features were identified through untargeted metabolomics (for putative annotations refer to **Supplemental Table 3**). Hierarchical clustering (Euclidean distance, ward linkage method) of the metabolite abundance is shown. Each row represents a metabolite and each column represents a patient. Metabolites in (A) marked with an (*) are putative.

**Supplemental Figure 2**. **Two-step process to identify a metabolite (example of anthranilate).** (A) Molecules are subjected to a chemical reaction by addition of a trimethylsilyl (TMS) functional group, injected into the gas chromatography (GC) column and adsorbed at its head. A temperature gradient applied to the column allows the separation of molecules with different boiling point, hence impacting their retention time (RT). Ionization of the molecule occurs in the source of the mass spectrometer (MS) with a triple quadrupole (QQQ) analyzer. The first quadrupole (Q1) selects a specific ion, fragments it in the second quadrupole (Q2), and a specific fragment ion is then detected by the third quadrupole (Q3). Association of the two m/z (mass-to-charge ratios) within the single reaction monitoring (SRM) transition at a pre-validated chromatographic retention time renders the ion signal of the molecule unique and specific. (B) Anthranilic acid standard molecule, eluting at a retention time of 13.4 min a 281.0 m/z ratio (Q1). Fragmentation of the precursor ion (Q2) results in formation of the 266.0 m/z product ion selected in Q3. Hence the SRM transition “281.0>266.0” at 13.4 min is specific to the anthranilic acid. Note that the standard is analyzed together with the biological samples for better retention time alignment. (C) Anthranilic acid SRM transition at 13.4 min in a critical COVID-19 sample (red trace) shows an increased ion signal as compared to the mild sample (blue trace).

**Supplemental Figure 3. Heatmap showing all the identified metabolites in COVID-19 patients receiving tocilizumab.** Metabolic signature obtained through targeted (A) and untargeted metabolomic analysis (B). Targeted analysis allowed the identification of 231 metabolites (A) in the baseline serum samples from COVID-19 patients who received tocilizumab treatment, while over 3500 metabolic features were identified through untargeted metabolomics (B) (for putative annotations refer to **Supplemental Tables 6**). Each row represents a metabolite and each column represents a patient. Hierarchical clustering (Euclidean distance, ward linkage method) of the metabolite abundance is shown. Metabolites in (A) marked with an (*) are putative.

**Supplemental Figure 4. Before tocilizumab infusion, circulating cytokines did not discriminate patients who evolved favorably and unfavorably after treatment.** Patients with baseline cytokines assessment in serum samples are represented (n=22). Wilcoxon rank-sum test was used to measures between controls, patients with favorable and unfavorable outcomes after tocilizumab infusion. *p<0.05, **p < 0.01, ***p < 0.001, ****p<0.0001.

**Supplemental Figure 5. Variance contribution plot.** Metabolites selected for their significance in the comparison between patients with favorable and unfavorable outcomes after tocilizumab infusion, contribute in a similar fashion to the PC1 variability, which allows for discrimination between groups.

**Supplemental Tables**

**Supplemental Table 1. Patient characteristics according to COVID-19 status from the non-interventional cohort.** Data presented are number (percentage) and mean (standard deviation). COVID-19: Coronavirus disease 2019; BMI: Body mass index; CRP: C-reactive protein; WHO: World health organization; CT-scan: computerized tomography scan; ECMO: Extracorporeal Membrane Oxygenation; Pa02: Partial Pressure of Oxygen; Fi02: Fraction of Inspired Oxygen; SOFA: Sequential Organ Failure Assessment
^#^The BMI was body mass (in kg) divided by the square of the body height (in m). ^¶^Non-parametric tests (chi-square test for nominal variables and Kruskall-Wallis test for interval variables) were performed for all comparisons in the table presented.

**Supplemental Table 2. Adjustment on comorbidities (including obesity, diabetes, hypertension and chronic kidney disease) of critical to mild COVID-19 patient metabolome differences.**

**Supplemental Table 3. All clean untargeted metabolomics from the non-interventional cohort.** Raw data obtained from the non-targeted metabolomic analysis showing tall the metabolic features identified in the plasma samples.

**Supplemental Table 4. Patients characteristics according to COVID-19 status from the interventional cohort.** Data presented are number (percentage) and mean (standard deviation). BMI: Body mass index; CRP: C-reactive protein; WHO: World health organization;
^#^The BMI was body mass divided by the square of the body height, resulting from mass in kilograms and height in meters. ^§^Based on the WHO international scale of clinical studies relating to COVID-19, have been established (10-point scale) (16). ^¶^Non-parametric tests (chi-square test for nominal variables and Kruskall-Wallis test for interval variables) were performed for all comparisons in the table presented.

**Supplemental Table 5. All clean targeted metabolomics from the non-interventional cohort.** Raw data obtained from the targeted metabolomic analysis showing tall the metabolic features identified in the plasma samples.

**Supplemental Table 6. All clean untargeted metabolomics from the interventional cohort.** Raw data obtained from the non-targeted metabolomic analysis showing tall the metabolic features identified in the plasma samples.

**Supplemental Table 7. All clean targeted metabolomics from the interventional cohort.** Raw data obtained from the targeted metabolomic analysis showing tall the metabolic features identified in the plasma samples.
