# Supplementary figures and images for: Metabolomic analyses of COVID-19 patients unravel stage-dependent and prognostic biomarkers
Source: Cell Death Dis. 2021 Mar 11;12(3):258. doi: 10.1038/s41419-021-03540-y (PMC7948172; doi:10.1038/s41419-021-03540-y)

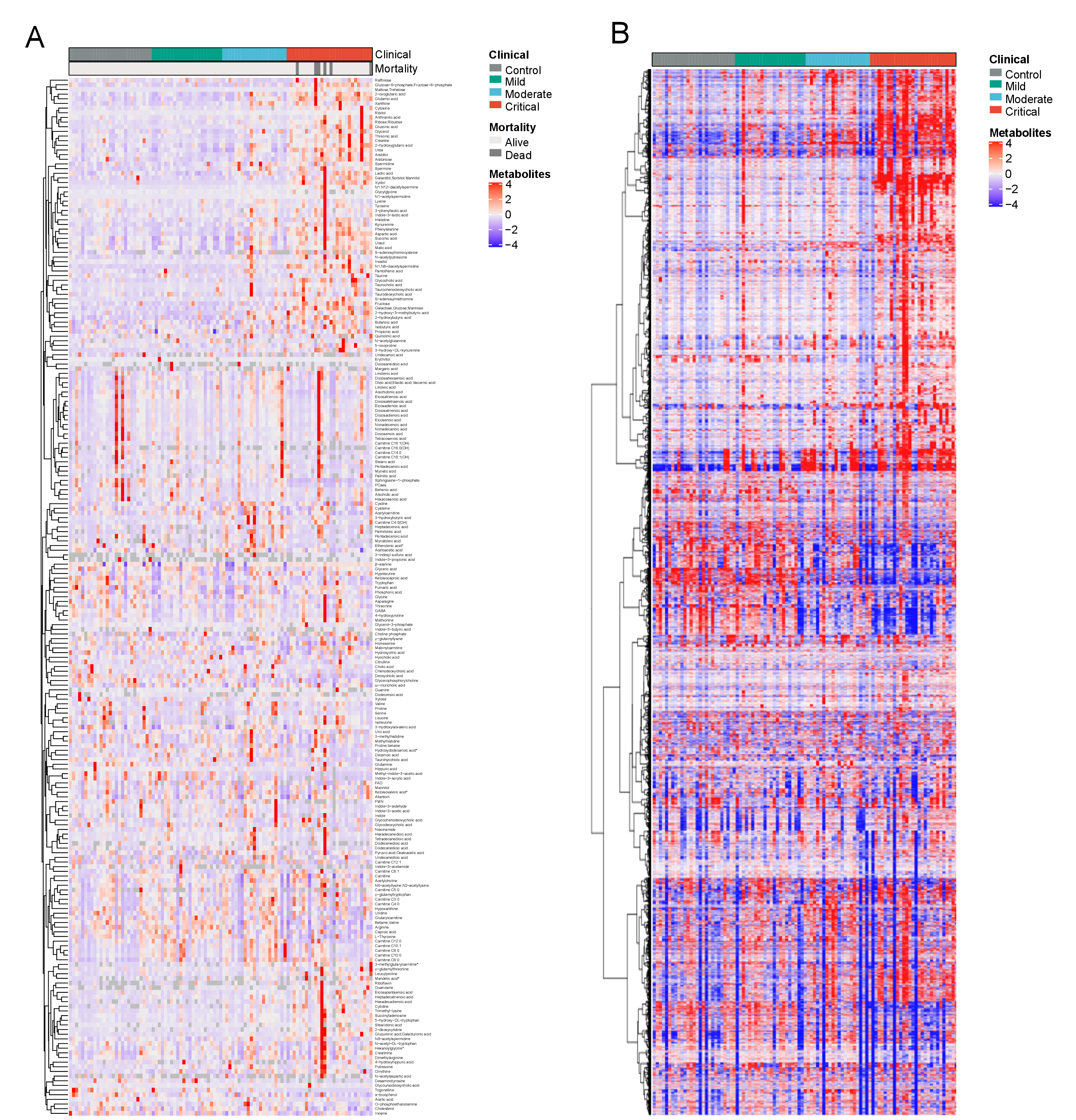

Supplement: Supplementary file 2 — Supplementary figure 1 [file 41419_2021_3540_MOESM2_ESM.tif]

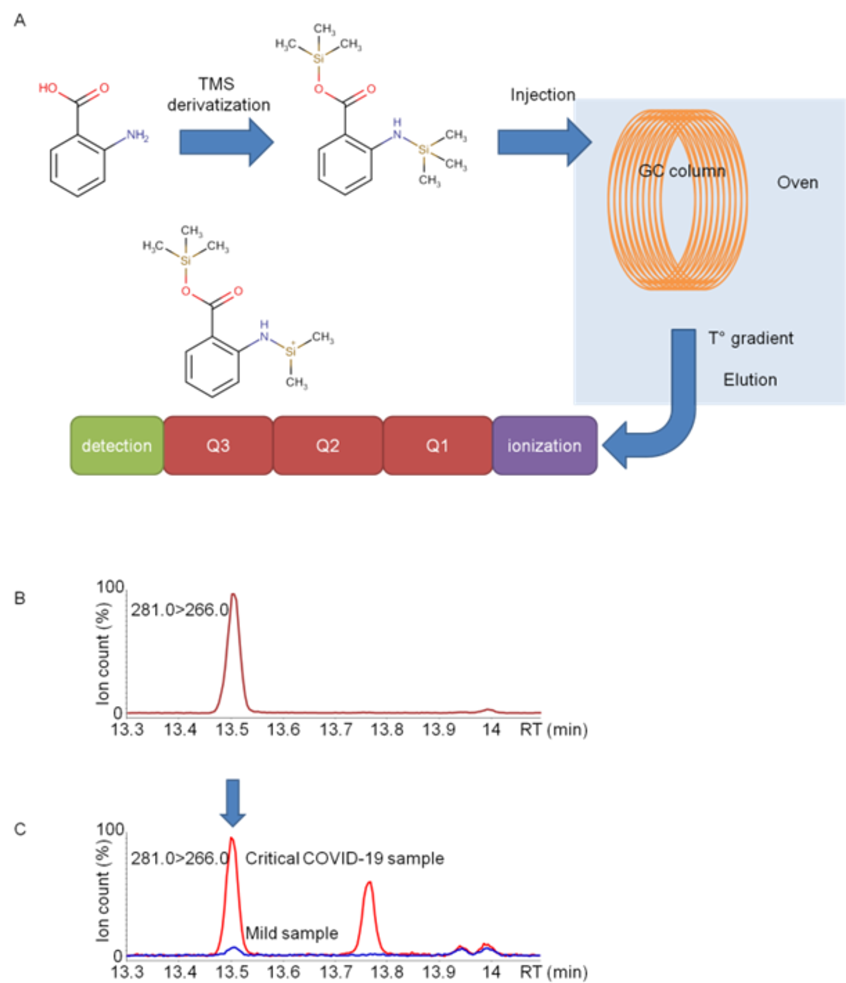

Supplement: Supplementary file 3 — Supplementary figure 2 [file 41419_2021_3540_MOESM3_ESM.tif]

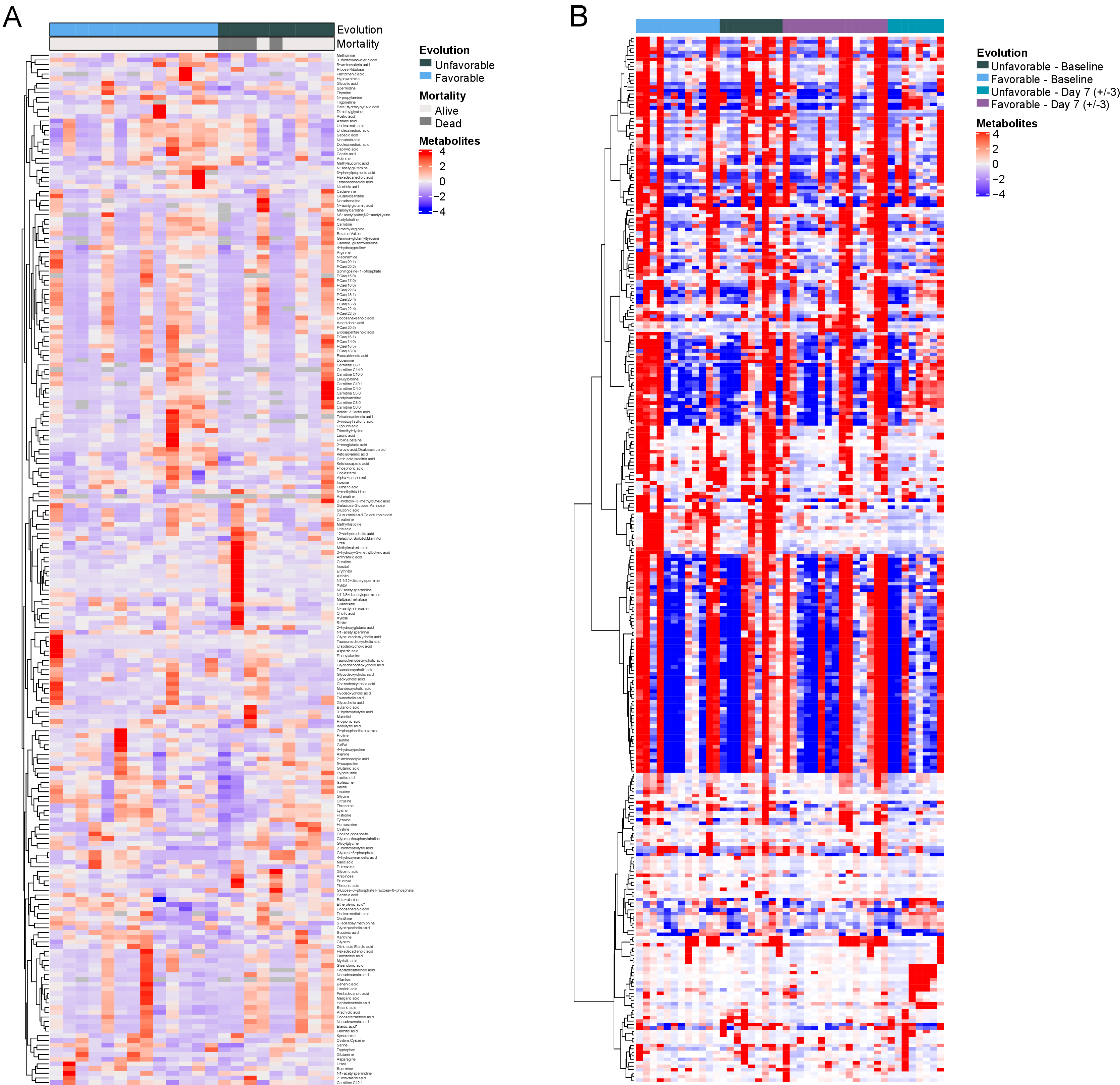

Supplement: Supplementary file 4 — Supplementary figure 3 [file 41419_2021_3540_MOESM4_ESM.tif]

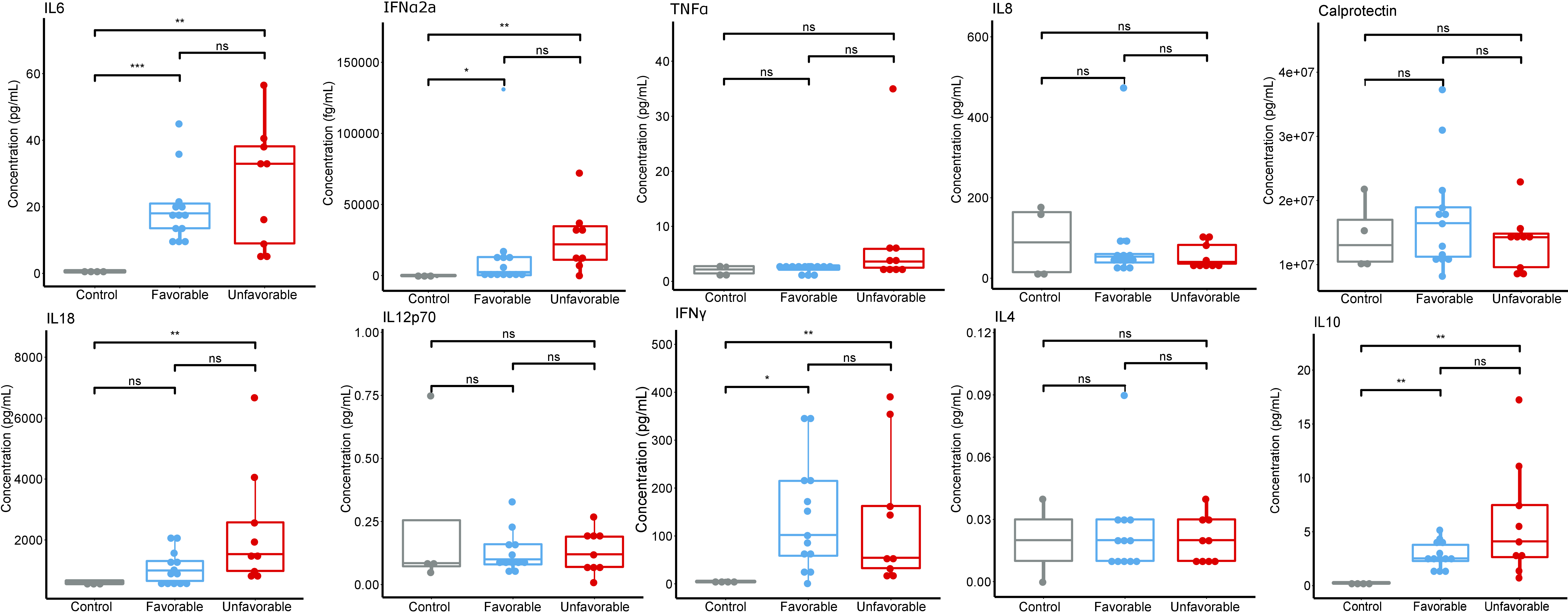

Supplement: Supplementary file 5 — Supplementary figure 4 [file 41419_2021_3540_MOESM5_ESM.tif]
