# Supplementary material for: Metabolomic analyses of COVID-19 patients unravel stage-dependent and prognostic biomarkers
Source: Cell Death Dis. 2021 Mar 11;12(3):258. doi: 10.1038/s41419-021-03540-y (PMC7948172; doi:10.1038/s41419-021-03540-y)
Supplement: Supplementary file 7 — Supplementary Table 1 [file 41419_2021_3540_MOESM7_ESM.docx]

| **Characteristics** | **Control**  **(n=27)** | **Mild**  **(n=23)** | **Moderate**  **(n=21)** | **Critical**  **(n=28)** | **p** |
| --- | --- | --- | --- | --- | --- |
|  |  |  |  |  |  |
| Gender |  |  |  |  |  |
| Female | 13 (48%) | 14 (61%) | 11 (52%) | 9 (32%) | 0.31 |
| Male | 13 (48%) | 9 (39%) | 9 (43%) | 19 (68%) |  |
| Age | 40 (13) | 40 (8.8) | 66 (17) | 57 (13) | <0.001 |
| BMI | 26 (4.6) | 23 (3.9) | 29 (4.8) | 29 (4) | <0.001 |
|  |  |  |  |  |  |
| **Comorbidities** |  |  |  |  |  |
| Hypertension | 3 (11%) | 1 (4.3%) | 9 (43%) | 12 (43%) | 0.001 |
| Dyslipidemia | 0 | 1 (4.3%) | 1 (4.8%) | 4 (14%) | 0.15 |
| Diabetes | 0 | 0 | 6 (29%) | 10 (36%) | <0.001 |
| Smoking | 8 (30%) | 9 (39%) | 2 (9.5%) | 4 (14%) | 0.062 |
| Chronic Kidney Disease | 0 | 0 | 1 (4.8%) | 5 (18%) | 0.017 |
| Cancer (all localized) | 0 | 1 (4.3%) | 0 | 3 (11%) | 0.15 |
| Hematological malignancy | 0 | 0 | 1 (4.8%) | 1 (3.6%) | 0.54 |
|  |  |  |  |  |  |
| **Clinical symptoms** | | | | | |
| Fever | 5 (19%) | 4 (17%) | 16 (76%) | 23 (82%) | <0.001 |
| Cough | 12 (44%) | 17 (74%) | 17 (81%) | 20 (71%) | 0.033 |
| Dyspnea | 8 (30%) | 5 (22%) | 8 (38%) | 22 (79%) | <0.001 |
| Myalgia | 15 (56%) | 12 (52%) | 7 (33%) | 9 (32%) | 0.2 |
| Diarrhea | 6 (22%) | 7 (30%) | 5 (24%) | 6 (21%) | 0.88 |
| Anosmia | 6 (22%) | 12 (52%) | 5 (24%) | 4 (14%) | 0.018 |
|  |  |  |  |  |  |
| **Biological characteristics** | | | | | |
| CRP (mg/l) | NA | 40 (16) | 73 (65) | 177 (85) | 0.0024 |
| Fibrinogen (g/L) | 3.1 (0.79) | 3.1 (0.9) | 5.3 (1.5) | 7 (1.8) | <0.001 |
| D-dimer (mcg/L) | 358 (332) | 528 (885) | 1800 (2300) | 4100 (3300) | <0.001 |
| Leucocytes (cell/mm^3^) | 7.3 (2.8) | 4.1 (1.3) | 6.1 (2.5) | 12 (10) | <0.001 |
| Neutrophils (cell/mm^3^) | 4 (2.7) | 2.5 (0.97) | 4.4 (1.9) | 7.8 (2.4) | <0.001 |
| Eosinophils (cell/mm^3^) | 0.14 (0.17) | 0.047 (0.05) | 0.14 (0.32) | 0.079 (0.09) | 0.21 |
| Basophils (cell/mm^3^) | 0.05 (0.024) | 0.023 (0.015) | 0.022 (0.02) | 0.64 (2.1) | <0.001 |
| Lymphocytes (cell/mm^3^) | 1.8 (0.58) | 1.1 (0.35) | 1.2 (0.63) | 0.97 (0.54) | <0.001 |
| Monocytes (cell/mm^3^) | 0.53 (0.28) | 0.41 (0.24) | 0.45 (0.24) | 0.42 (0.4) | 0.3 |
| Hemoglobins (cell/mm^3^) | 14 (1.3) | 12 (4.8) | 13 (1.8) | 10 (2.2) | <0.001 |
| Platelets (cell/mm^3^) | 251 (56) | 178 (42) | 256 (102) | 245 (89) | 0.15 |
|  |  |  |  |  |  |
| **Chest CT characteristics** | | | | | |
| Pneumonitis (CT scan) | 0 | 1 (4.3%) | 19 (90%) | 23 (82%) | <0.001 |
| <10% | 0 | 1 (4.3%) | 3 (14%) | 0 | 0.046 |
| 10-24% | 0 | 0 | 9 (43%) | 4 (14%) | <0.001 |
| 25-50% | 0 | 0 | 7 (33%) | 6 (21%) | 0.0018 |
| >50% | 0 | 0 | 0 | 7 (25%) | <0.001 |
|  |  |  |  |  |  |
| **Therapeutic interventions** |  |  |  |  |  |
| Oxygenotherapy | 0 | 0 | 18 (86%) | 28 (100%) |  |
| Non Invasive Ventilation | 0 | 0 | 16 (76%) | 9 (32%) |  |
| Orotracheal Intubatuion | 0 | 0 | 0 | 23 (82%) |  |
| Ventral Decubitus maneuver | 0 | 0 | 0 | 15 (54%) |  |
| ECMO | 0 | 0 | 0 | 4 (14%) |  |
| P_a_02F/Fi02 (at sampling) |  |  |  | 154 (51) |  |
| Respiratory SOFA |  |  |  | 3 (0.47) |  |
| General SOFA |  |  |  | 8.3 (3.5) |  |
|  |  |  |  |  |  |
| Hydroxychloroquine | 0 | 0 | 1 (4.8%) | 6 (21%) | 0.01 |
| Azythromycine | 0 | 0 | 3 (14%) | 8 (29%) | 0.0034 |
| Antibiotics | 0 | 1 (4.3%) | 5 (24%) | 23 (82%) | <0.001 |
| Curative anticoagulation | 0 | 0 | 9 (43%) | 16 (57%) | <0.001 |
| Preventive anticoagulation | 0 | 0 | 4 (19%) | 10 (36%) | 0.0015 |
| Prednisone | 0 | 0 | 2 (9.5%) | 2 (7.1%) | 0.35 |
| Anakinra | 0 | 0 | 1 (4.8%) | 0 | 0.42 |
| Sarilumab | 0 | 0 | 1 (4.8%) | 2 (7.1%) | 0.47 |
| Corimmuno trial | 0 | 0 | 3 (14%) | 2 (7.1%) | 0.17 |
| Remdesivir | 0 | 0 | 0 | 7 (25%) | 0.0018 |
| Lopinavir/ritonavir | 0 | 0 | 2 (9.5%) | 0 | 0.13 |
|  |  |  |  |  |  |
| **Outcomes** |  |  |  |  |  |
| Extubation |  |  |  | 16 (57%) |  |
| Discharged from ICU |  |  |  | 21 (75%) |  |
| Discharged from Hospital |  |  | 17 (81%) | 16 (57%) | <0.001 |
| Death | 0 | 0 | 1 (4.8%) | 5 (18%) | <0.001 |
