# Supplementary material for: Metabolomic analyses of COVID-19 patients unravel stage-dependent and prognostic biomarkers
Source: Cell Death Dis. 2021 Mar 11;12(3):258. doi: 10.1038/s41419-021-03540-y (PMC7948172; doi:10.1038/s41419-021-03540-y)
Supplement: Supplementary file 10 — Supplementary Table 4 [file 41419_2021_3540_MOESM10_ESM.docx]

|  | **Favorable**  **(n=16)** | **Unfavorable**  **(n=9)** | **p** |
| --- | --- | --- | --- |
|  |  |  |  |
|  |  |  |  |
| Gender |  |  |  |
| Female | 2 (12%) | 1 (11%) |  |
| Male | 14 (88%) | 8 (89%) |  |
| Age | 54 (12) | 59 (8.9) | 0.35 |
|  |  |  |  |
| **Comorbidities** | | |  |
| Overweigh (BMI>30) | 3 (19%) | 3 (33%) | 0.24 |
| Chronic Respiratory Disease | 2 (12%) | 1 (11%) | 1 |
| Chronic Kidney Disease | 0 | 1 (11%) | 0.77 |
| Chronic Heart Disease | 2 (12%) | 0 | 0.74 |
| Hypertension | 2 (12%) | 3 (33%) | 0.47 |
| Diabetes | 0 | 4 (44%) | 0.019 |
| Cancer | 2 (12%) | 2 (22%) | 0.95 |
| Hematological malignacy | 0 | 2 (22%) |  |
|  |  |  |  |
| **Clinical presentation at baseline samples** | | | |
| Delay between first symptoms and tocilizumab infusion | 10 (3.2) | 9.6 (4.4) | 0.77 |
| WHO Progression scale | 5 (0) | 5 (0) | 1 |
| Respiratory rate (.min^-1^) | 29 (6.8) | 27 (6.4) | 0.89 |
| SaO2 (%) | 92 (4.8) | 94 (1.9) | 0.39 |
| Oxygenotherapy (L.min^-1^) | 11 (3.3) | 9.4 (2.7) | 0.77 |
| Temperature (°c) | 38 (1.2) | 38 (0.75) | 0.44 |
| CRP (mg/L) | 187 (105) | 173 (76) | 0.78 |
| Lymphocytes (cell/mm^3^) | 831 (320) | 433 (339) | 0.057 |
| Neutrophils (cell/mm^3^) | 5800 (2400) | 5500 (4400) | 0.47 |
| Monocytes (cell/mm^3^) | 438 (247) | 389 (183) | 1 |
| Eosinophils | 44 (109) | 0 (0) | 0.99 |
| LDH (UI/mL) | 518 (220) | 423 (64) | 0.16 |
| D-dimer (mcg/L) | 3600 (6000) | 2000 (3400) | 0.8 |
| PCT (mcg/L) | 0.66 (1.4) | 3.5 (7) | 0.82 |
| Ferritin (mcg/L) | 1900 (1200) | 1700 (1200) | 1 |
| Fibrinogen (g/L) | 6.1 (2) | 6.3 (1.7) | 0.72 |
|  |  |  |  |
|  |  |  |  |
|  |  |  |  |
| **Therapeutic interventions** | | |  |
| Second tocilizumab infusion | 10 (62%) | 4 (44%) | 0.65 |
| Lopinavir/Ritonavir | 2 (12%) | 1 (11%) | 1 |
| Azithromycine | 12 (75%) | 6 (67%) | 1 |
| Hydroxychloroquine | 5 (31%) | 4 (44%) | 0.82 |
| Corticosteroids (>20mg/d prednisone) | 3 (19%) | 4 (44%) | 0.36 |
| WHO progression scale at day 7 |  |  | 0.0016 |
| 4 | 3 (19%) | 0 |  |
| 5 | 13 (81%) | 2 (22%) |  |
| 6 | 0 | 1 (11%) |  |
| 7/8/9 | 0 | 4 (44%) |  |
| 10 (dead) | 0 | 2 (22%) |  |
| Admission in ICU | 2 (12%) | 7 (78%) | 0.0047 |
| Orotracheal Intubation | 0 | 4 (44%) | 0.019 |
| Non-Invasive Ventilation | 2 (12%) | 5 (56%) | 0.066 |
| Oxygenotherapy (L.min^-1^) | 5.2 (4.2) | 10 (2.8) | 0.27 |
| Temperature (°c) | 37 (0.44) | 37 (0.89) | 0.12 |
| Respiratory rate (/min) | 24 (5) | 30 (9.8) | 0.17 |
| CRP (mg/L) | 4.5 (2.4) | 25 (30) | 0.012 |
|  |  |  |  |
| **Outcomes** |  |  |  |
| Follow-up (days) | 18 (13) | 21 (16) | 0.92 |
| Thrombosis | 19% (3) | 11% (1) | 1 |
| Death | 0% (0) | 44% (4) | 0.019 |
|  |  |  |  |
